# Remote noninvasive allograft rejection monitoring for heart transplant recipients: study protocol for the novel evaluation with home electrocardiogram and remote transmission (NEW HEART) study

**DOI:** 10.1186/1471-2261-12-14

**Published:** 2012-03-02

**Authors:** Lynn V Doering, Kathleen Hickey, David Pickham, Belinda Chen, Barbara J Drew

**Affiliations:** 1 University of California, Los Angeles (UCLA), School of Nursing, 700 Tiverton Ave., Factor Building 4-266, Los Angeles, Ca 90095, USA; 2 Columbia University, School of Nursing, 630 West 168th Street, New York, NY 10032, USA; 3 University of California, San Francisco (UCSF), 2 Koret Way, N611H, San Francisco, CA 94143-0610, USA; 4 University of California, Los Angeles (UCLA), School of Nursing 700 Tiverton Ave. Factor Building 4-266, Los Angeles, Ca 90095, USA; 5 University of California, San Francisco (UCSF), 2 Koret Way, N611H, San Francisco, CA 94143-0610, USA

**Keywords:** ECG monitoring, QT interval, Heart transplantation, Allograft rejection

## Abstract

**Background:**

Acute allograft rejection is a major cause of early mortality in the first year after heart transplantation in adults. Although endomyocardial biopsy (EMB) is not a perfect "gold standard" for a correct diagnosis of acute allograft rejection, it is considered the best available test and thus, is the current standard practice. Unfortunately, EMB is an invasive and costly procedure that is not without risk. Recent evidence suggests that acute allograft rejection causes delays in ventricular repolarization and thereby increases the cellular action potential duration resulting in a longer QT interval on the electrocardiogram (ECG). No prospective study to date has investigated whether such increases in the QT interval could provide early detection of acute allograft rejection. Therefore, in the Novel Evaluation With Home Electrocardiogram And Remote Transmission (NEW HEART) study, we plan to investigate the potential benefit of daily home QT interval monitoring to predict acute allograft rejection.

**Methods/design:**

The NEW HEART study is a prospective, double-blind, multi-center descriptive research study. A sample of 325 adult heart transplant recipients will be recruited within six weeks of transplant from three sites in the United States. Subjects will receive the HeartView™ ECG recorder and its companion Internet Transmitter, which will transmit the subject's ECG to a Core Laboratory. Subjects will be instructed to record and transmit an ECG recording daily for 6 months. An increase in the QT_C _interval from the previous day of at least 25 ms that persists for 3 consecutive days will be considered abnormal. The number and grade of acute allograft rejection episodes, as well as all-cause mortality, will be collected for one year following transplant surgery.

**Discussion:**

This study will provide "real world" prospective data to determine the sensitivity and specificity of QT_C _as an early non invasive marker of cellular rejection in transplant recipients during the first post-transplant year. A non-invasive indicator of early allograft rejection in heart transplant recipients has the potential to limit the number and severity of rejection episodes by reducing the time and cost of rejection surveillance and by shortening the time to recognition of rejection.

**Trial Registration:**

ClinicalTrials.gov: NCT01365806

## Background

The prevalence of American adults living with a heart transplant was 20,369 in 2009, the most recent year for which complete data are available [1]. Acute allograft rejection is a major cause of early mortality, a rate that reaches 13% in the first year after heart transplantation in adults [1,2]. According to the 2011 annual United States data published from the International Society for Heart Lung Transplantation Registry, 26% of heart transplant recipients have at least one rejection episode within the first year following transplant surgery [2]. Acute rejection remains the most common cause of morbidity and rehospitalization. Jalowiec [3] reported that 64% of heart transplant recipients were rehospitalized during the first year after transplant surgery (median length of stay, 16 days), and 37% were rehospitalized more than once. Rejection is also a primary cause of urgent re-transplantation, a situation that is perceived by some to be morally unfair because these patients are allowed a second transplant while others are waiting and often dying before receiving their first transplant. Thus, the financial, physical, and emotional toll associated with acute rejection is significant.

In order to detect the early stages of rejection so that more aggressive and early immunosuppressant therapy can be initiated, frequent biopsies of heart tissue are performed (typically, weekly or every other week in the first three months and then monthly or every other month during the first year). Although endomyocardial biopsy (EMB) is not a perfect "gold standard" for a correct diagnosis of acute allograft rejection, it is considered the best available test and thus, it is the current standard practice. Unfortunately, EMB is an invasive and costly procedure that is not without risk [4,5]. If a simple noninvasive biomarker could be identified to detect the early stages of acute rejection, it might be possible to reduce the number of invasive biopsy procedures and to initiate earlier therapy that might prevent death from severe rejection.

Alternatives to invasive EMB monitoring have been the subject of recent study. Most prominently, the use of a commercially available test, the AlloMap, that determines gene-expression profiling of recipient leukocytes, was tested against routine EMB to determine whether adverse events (a composite of allograft dysfunction, death, or retransplantation) differed between patients who received standard EMB monitoring and those who received monitoring by gene-expression profiling [6]. Although the authors concluded that the gene-expression profiling was not inferior to EMB in its association with adverse events, only 6 of 34 rejection episodes in the AlloMap group were identified solely on the basis of the profiling test [6]. Further, the wide confidence intervals for adverse events translated into as much as a 68% increase in risk with AlloMap monitoring [7]. Most participants were randomized more than one year after transplant, which limits the generalizability of these findings to the higher risk, early (< one year) post-transplant group that we propose to study.

Other investigators have focused on the electrocardiogram (ECG) as a potential marker of allograft rejection because it is noninvasive and easily measured. Previous studies have investigated the QT interval, an indirect measure of the cellular action potential duration of ventricular myocytes. The QT interval, defined as the interval from the beginning of the QRS complex to the end of the T wave, reflects the time that elapses between the initial fast depolarization of the ventricles and their subsequent repolarization. Recent evidence suggests that acute allograft rejection causes delays in ventricular repolarization and thereby increases the cellular action potential duration resulting in a longer QT interval on the ECG [8-12]. Findings from two studies suggest that the QT interval is linked to acute allograft rejection and three other studies have linked an increased QT interval with mortality (Table 1). However, these studies all have serious limitations. All but one were retrospective analyses. All involved hospital or clinic-acquired ECGs, typically done at baseline seven days following transplant surgery and then annually or at the time of hospitalization for acute severe allograft rejection. Thus, none of the studies could report whether QT interval prolongation was an early sign of rejection or simply the result of severe, irreversible rejection. Another limitation was that all but one study made no mention of investigators being blinded from clinical information about allograft rejection. Thus, it is possible that the researchers making manual measurements of the QT interval were biased by knowing whether the subject did or did not have acute allograft rejection. Despite these limitations, there is evidence that an increase in the QT interval is linked to acute allograft rejection and mortality.

**Table 1:** Human Studies on QT Interval Prolongation after Heart Transplantation

# Subjects Author/Yr	Study Design	Major Findings	Study Limitations	Implications for NEW HEART Study
N = 65 Richartz et al. 1998 [8]	Prospective; Rejection during in-hospital period	1. Mean QT_C _= 449 ± 2 ms without rejection; 517 ± 11 ms with rejection (*p *< 0.001) 2. > 10% increase in QT_C _predicted rejection with sensitivity, 86%; specificity, 88%	1. Only observed rejection during hospitalization for transplant surgery 2. Analyzed only 3 ECGs per subject 3. ECG analyzers not blinded from clinical information about rejection	An increase in the QT interval predicts acute allograft rejection

N = 52 Kolasa et al. 2005 [9]	Retrospective; Long-term mortality over 7 yrs	Poorer survival over 7 yrs in subjects who had a > 10 ms per year change in their QT_C _interval on their annual ECG	1. Link to rejection not studied 2. Analyzed only one ECG per year	As little as a 10 ms change in the QT interval is linked to mortality

N = 200 Tenderich et al. 2006 [10]	Retrospective; Rejection during first 3 months	> 25 ms increase in QT_C _interval predicted acute allograft rejection with sensitivity, 77%; specificity, 96%.	1. Only analyzed 2 ECGs per subject 2. ECG analyzers not blinded from clinical information about rejection	Increase in QT interval predicts acute rejection during the period of interest

N = 587 Vrtovec et al. 2006 [11]	Retrospective; Long-term mortality up to 17 yrs	1. Patients with ≥ 10% increase in QT_C _interval between 1^st ^and 2^nd ^year post-transplant had 6.86 times higher risk of dying.2. ≥ 10% increase in QT_C _was the only independent predictor of long- term mortality on multivariate analysis 3. Trend was a decreasing QT_C _over the yrs suggesting sympathetic reinnervation	1. Link to rejection not studied 2. Analyzed only one ECG per year	An increase in the QT interval is linked to mortality

N = 71 Vrtovec et al. 2008 [12]	Retrospective; 1- year all-cause & SCD mortality in subjects with severe acute rejection	1. QT_C _was longer in SCD group than in survivors (475 ± 57 versus 437 ± 36 ms; *p *= 0.02) 2. Patients who had > 10% increase in QT interval during a severe acute rejection episode were at increased risk for SCD.	1. Limited to just patients with severe rejection; unable to tell whether increased QT interval could predict earlier, milder forms of rejection 2. Analyzed just 2 ECGs per subject (one at baseline 7 days after surgery, second at time of rejection)	An increase in the QT interval is linked to mortality

ms = millisecond; SCD = sudden cardiac death; QTc = QT interval corrected for heart rate

No prospective study to date has investigated whether such increases in the QT interval could provide early detection of acute allograft rejection. In addition, advances in home ECG monitoring have evolved and become less cumbersome and more patient-friendly. Therefore, in the Novel Evaluation With Home Electrocardiogram And Remote Transmission (NEW HEART) study, we plan to take advantage of novel ECG technology to investigate the potential benefit of daily home QT interval monitoring to predict acute allograft rejection following heart transplantation.

## Methods/design

### Design

The NEW HEART study is a prospective, double-blind, multi-center descriptive research study. The primary aims of the NEW HEART study are to: 1) determine whether an increase in the QT interval during the first 6 months following heart transplant is a sensitive and specific biomarker for acute allograft rejection; 2) determine the timing of initial increased QT interval relative to biopsy-diagnosed stages of mild, moderate, and severe allograft rejection.

Secondary aims are to: 1) determine whether an increase in the QT interval during the first 6 months following heart transplant predicts mortality within the first year, and 2) explore additional ECG measurements that might predict acute allograft rejection or death.

### Sample

A sample of 325 adult heart transplant recipients will be recruited from the Columbia University-New York Presbyterian Medical Center (CU-NYP), the University of California, Los Angeles (UCLA), and Cedars Sinai Medical Center (CSMC) in Los Angeles, CA. Respectively, demographic characteristics at these sites are: male (68.9%, 75.7%, and 79.7%); White (53.1%, 59.1%, 73.4%); Black (20.3%, 9.4%,12.5%); Latino (14.5%, 19.9%, 9.4%) and Asian (11.3%, 10.5%, 3.1%) [1]. Inclusion criteria are: 1) ≥ 18 years of age, 2) first heart transplant surgery within six weeks of their transplant surgery, 3) not enrolled in other research studies that conflict with the study design, 4) clinically stable at time of enrollment (i.e. no clinical symptoms of allograft impairment with ejection fraction ≥ 45%).

### Instrumentation

QT interval was selected for monitoring in this study because it is the ECG element most likely to reflect early acute rejection, as previously noted, and because in the setting of early post-transplantation, it is free of neurologically mediated influences. In the first year after transplantation, the cardiac allograft is denervated [13]. A potential major benefit of allograft denervation in the NEW HEART study is that without the confounding influences of heart rate and autonomic nervous system activity, an observed increase in the QT interval is likely to indicate abnormal ventricular repolarization due to another cause, such as acute allograft rejection.

After a thorough search of the available technology, the HeartView™ ECG Personal Recorder-Transmitter (Aerotel Medical, Israel) was selected. The ECG device records 10 s of the six limb leads (I, II, III, aVR, aVL, aVF) and two precordial leads (V5 and V6 will be used for this study). The HeartView™ device acquires data at a sampling rate of 500 samples per second which is the standard resolution recommended for diagnostic electrocardiography. The device's reliability and validity for ECG diagnosis are evidenced by approval by the U.S. Food & Drug Administration that requires the device to meet criteria stipulated by the Association for the Advancement of Medical Instrumentation for electrocardiograph instruments [14].

**Figure 1:**
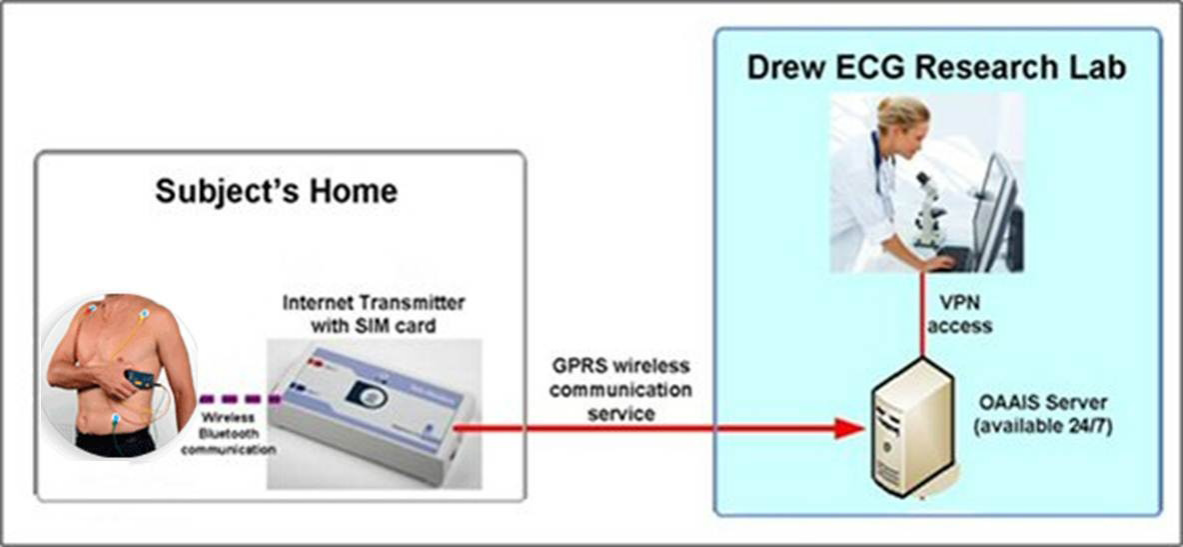
**HeartView™ Device and Transmission System**.

The HeartView™ will be used to transmit the subject's ECG to the Core Laboratory at UCSF ("Drew Lab") (Figure 1). Subjects will be given HeartView™ and Internet Transmitter devices (Aerotel Medical, Israel) for the 6-month monitoring period. Once the patient records his ECG, the HeartView™ device will automatically seek, find, and upload the ECG by wireless Bluetooth communication to the Internet Transmitter. Then, using mobile phone technology (subscriber identity module [SIM] card), the Internet Transmitter device will automatically seek, find, and send the digital ECG to a UCSF server via wireless General Packet Radio Services (GPRS) internet access. Thus, subjects do not have to dial a telephone to transmit the ECG, nor do they need to have a home computer or even be computer literate. Subjects need only to record their ECG; the rest will be automatic.

### Procedures

Participants will be recruited either while they are in the hospital for transplant surgery, or if that is inconvenient, during their first clinic visit following transplant surgery. After informed consent is obtained, demographic and clinical data will be collected by self-report and by medical record audit. At the time of each EMB, data from the medical record will be abstracted to confirm current medications, serum levels of anti-rejection medications, clinical data regarding EMB results, and other clinical data reflecting myocardial performance consistent with rejection status. Only one anti-rejection medication (tacrolimus) is associated with an increased QT interval. If a patient is on chronic tacrolimus therapy without a change in dosage, it should not interfere with the daily ECG monitoring measurement because we will be comparing each patient with him/herself and noting any change from the previous day's QT interval. However, we will track initiation or change in any QT-prolonging drug. On their website, the Arizona Center for Education and Research on Therapeutics [15] maintains a comprehensive list of QT- prolonging drugs that we have incorporated into the medications section of our electronic database system. Further, on a quarterly basis, we will continue to reference this website for new drugs as they come to market and update our data capture to include those associated with QT-prolongation.

The other drug class that patients may be taking is 3-Hydroxy-3-Methyl-Glutaryl Coenzyme A Reductase inhibitors (i.e., statins) which are known to shorten the QT interval. Thus, we will also monitor statin therapy. To insure patient safety, we will notify patients' transplant cardiologists within 48 h of the detection of any QTc interval exceeding the population 99th percentile for adult males (> 470 ms) or females (> 480 ms) [16].

At the time of informed consent and enrollment, subjects will receive the HeartView™ recorder and its companion Internet Transmitter, which will transmit the subject's ECG as described earlier. Subjects will be asked to record a 10-s 8-lead ECG daily for 6 months.

Participants and their caregivers, if available, will be instructed in the use of the HeartView device using a standardized training booklet. Each enrollee will be given the opportunity to practice transmission and will receive feedback, if needed, prior to the beginning of daily recordings. Daily recordings will be monitored by study personnel to insure quality of transmissions.

Participants will be instructed to make and upload ECG recordings at any time of day that is convenient for them, although they will be urged to select a consistent time to promote adherence to recording. Across participants, a standard recording time is not required because in the early post transplant period complete allograft denervation results in failure of parasympathetic or sympathetic nerves to influence heart rate. In normal individuals at rest, parasympathetic influences via the vagus nerve predominate resulting in a resting heart rate of 60-90 beats/min in adults. However, without such parasympathetic influence on the allograft, transplant recipients experience a permanent tachycardia (99 ± 12 beats/min) and exhibit little heart rate variability over a 24 h period [17]. For the current study, this means that subjects will be able to record their daily ECGs at any time without the confounding influence of 24-h variability on ECG intervals. However, participants will be instructed to wait for at least ten minutes after exercising before recording an ECG. This is necessary because allograft beta receptors are functional early after transplant [17] and respond to increases in circulating catecholamines, such as that elicited by exercise. This post-transplant response to exercise is less immediate than in normal individuals and after cessation of exercise, peak heart rate also recedes at a slower pace, over about a 10-min period [17].

### Analysis of ECG monitoring data

The ECG will be sent to a large server hosted by the UCSF Office of Academic and Administrative Information Systems (OAAIS). The server is a secure, highly redundant data center with 24/7 availability due to back-up generators that protect against power outages. The system provides nightly data backup. Investigators in the Drew Lab will access the ECG data by logging on with a username and password via a Virtual Private Network (VPN), which encapsulates data transfers between networked devices not on the same private network and keeps the transferred data private from other devices. Investigators who analyze the daily ECG data will be blinded from all information regarding the subject's clinical status and biopsy results. In addition, pathologists who grade the biopsies and clinicians who provide medical care in the transplant clinics will be blinded from the QT interval monitoring data.

**Figure 2:**
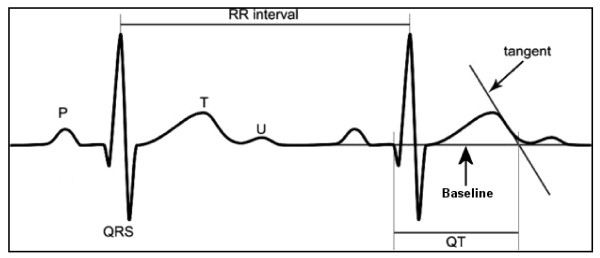
**Measurement of QT Interval**. A tangent is drawn to the steepest slope of the last limb of the T wave; the end of the T wave is the intersection of this tangent with the baseline.

QT and RR intervals will be made in a computer-assisted manner. The Aerotel measurement software provides a zooming feature to enlarge ECG waveforms for better visualization. In addition, electronic calipers are provided so that the researcher will select the appropriate waveform onset and offset points and the computer software will provide the interval value in milliseconds (ms). The QT interval will be measured from the onset of the QRS waveform to the end of the T waveThe end of the T wave will be defined as the intersection of a tangent to the steepest slope of the last limb of the T wave and the baseline (Figure 2) [18]. A standardized correction to account for the effect of HR will be applied (QT_C_). An increase in the QT_C _interval from the previous day of at least 25 ms that persists for 3 consecutive days will be considered abnormal [10].

To explore the potential diagnostic and prognostic value of additional ECG measurements, the following variables will also be measured on the daily ECG recordings: cardiac rhythm, heart rate, P wave and QRS duration, PR interval, QRS amplitude, and the interval from the peak of the T wave to the end of the T wave [19]. A prolonged T_PEAK _- T_END _interval is thought to represent heterogeneity of ventricular repolarization across the 3 layers of the myocardial wall [20] and has been associated with risk for sudden cardiac death from torsades de pointes [21].

### Diagnosis of acute allograft rejection

Rates of acute allograft rejection have varied, but the most recent rates of rejection in the first 6 months after transplant are reported to be 25 to 35% [2]. The diagnosis of acute allograft rejection is made by an EMB which will be performed in the hospital cardiac catheterization laboratory at each site. Generally, 3-6 biopsy specimens of the right ventricular endocardium are taken to minimize false negative tests. Each site will follow its own acute rejection surveillance protocol. In general, these protocols require weekly or biweekly EMBs during the first two to three post-transplant months. After that time, biopsies are performed less frequently, based on clinical judgment and risk of rejection. At each site, EMBs will be graded by clinical pathologists experienced with the heart transplant population and using the International Society of Heart and Lung Transplantation standards for acute rejection. Although early reports indicated inter-operator variability in the interpretation of EMB results [22], the use of the standardized grading system has improved the reliability and validity of the measurement. Current variability in interpretation of biopsy results occurs mainly in the diagnosis of Grade 2 rejection by local pathologists which may be misinterpreted in the presence of endocardial infiltrates called Quilty lesions [23,24]. To control for potential variability, we will monitor and record clinical and hemodynamic indicators of myocardial performance, which are obtained at the time of each biopsy or at regular clinical assessments, to support the biopsy interpretation, particularly regarding Grade 2 rejection. Such a multi-factorial approach to identification of rejection has been recommended [24].

### Follow-up and end-points

The number and grade of acute allograft rejection episodes will be collected for a period of one year following transplant surgery. Data on all-cause mortality within the first year will be collected. Because all transplant centers are required to report their data to the transplant registry, we anticipate no loss to follow-up or missing data regarding rejection or death.

### Data management

To insure accuracy of data collection, all sites will use a secure web-based data capture site, Research Electronic Data Capture (REDCap) [25], which is sponsored by a consortium of 118 research institutions. REDCap offers a stream-lined process for rapidly building a database, an interface for data collection and validation, and automated export procedures for download to statistical packages. As part of our pilot study, we developed, initiated, and refined a REDCap database which is ready for use in the proposed study.

### Statistical analysis

Using the variable definitions in Table 2, a logistic regression analysis will be performed with the independent variable being presence/absence of the ECG criteria (ΔQT_C _≥ +25 ms × 3 days [10]) and the dependent variable being presence/absence of acute allograft rejection. Odds ratios and confidence intervals will be reported as well as the universal proportions used to determine the value of new diagnostic criteria (sensitivity, specificity, positive/negative predictive value, and predictive accuracy, Table 3).

**Table 2:** Variable definitions for statistical analysis

Variable	Definition	Type of Variable
ΔQT_C_	≥ +25 ms increase in QT_C _interval from previous day lasting 3 consecutive days [10]	Continuous (expected range, 10-100 ms)

Acute allograft rejection	EMB category 1R or 2R or 3R with confirmation by treating physician	Categorical; dichotomous (yes/no) No = 0; Yes = 1R or 2R or 3R

Days to rejection	Number of days from first positive ECG criterion to mild, moderate, or severe rejection diagnosis. If the ECG criterion follows rejection, a negative # of days will be reported.	Continuous (expected range -365 to +365)

Acute rejection severity	EMB category: 1R (mild), 2R (moderate), 3R (severe)	Categorical

Cardiac rhythm	Sinus rhythm, atrial fibrillation, atrial flutter, junctional rhythm	Categorical

Heart rate	Heart rate per minute calculated from 30 s rhythm strip	Continuous (expected range, 60-120)

P wave duration	Interval from beginning to end of P wave in ms (measure of intra-atrial conduction delay)	Continuous (expected range, 70-126 ms)

QRS duration	Interval from beginning to end of the QRS waveform	Continuous (expected range, 72-160 ms)

PR interval	Interval from the beginning of the P wave to the beginning of the QRS complex measured in ms. PR interval > 200 ms indicates an abnormal delay of conduction (1^st ^degree AV block).	Continuous (expected range, 112-240 ms)

QRS amplitude	Height of QRS waveform in μV	Continuous (expected range, 500-1000 μV

T_PEAK _- T_END_	Interval from the peak of the T wave (or nadir in inverted T waves) to the end of the T wave in ms. Prolonged T_PEAK _-T_END _indicates heterogeneity of repolarization and risk for arrhythmia	Continuous (expected range, 112-240 ms)

**Table 3:** Definitions of Universal Proportions

Criterion	Definition
Sensitivity	Proportion of those with acute rejection who are positive for the ECG criterion

Specificity	Proportion of those without rejection who are negative for the ECG criterion

Positive Predictive Value	Proportion of patients with positive ECG criterion who have rejection

Negative Predictive Value	Proportion of patients with negative ECG criterion who do not have rejection

Predictive Accuracy	$\frac{\#\text{true positives}\pm \text{true negatives by the ECG criterion}}{\text{Total }\#\text{ of patients enrolled in the study }\times \text{ 1}00}$

Descriptive statistics (means ± SD) will be used to describe the average time period between the development of the ECG criterion and biopsy evidence of rejection. In addition, we will report the proportion of subjects who develop the ECG criterion (≥ +25 ms increase in QT interval from previous day lasting 3 consecutive days [10]) before, during, or after the stages of mild, moderate, and severe rejection.

The Kaplan-Meier method with the log-rank test will be used to estimate survival curves over one year and to compare all-cause mortality in patients who are positive or negative for the QT_C _criterion. Cox Proportional Hazard modeling will be used to control for baseline differences between groups and for tacrolimus use.

Logistic regression will be used with the dependent variables of rejection (yes/no) and all- cause mortality (yes/no). In both regressions, tacrolimus dosage (with "0" entered if the patient is not receiving tacrolimus) will be included as a covariate in the model. If any of the explorative variables are statistically significant, a multiple logistic regression model will be used including the ECG criterion of interest to determine whether additional ECG criteria are independent predictors of rejection and death.

### Ethics approval

Institutional Review Board (IRB) approvals have been obtained at all participating sites. Although this is not a randomized clinical trial, we are implementing an external Data Safety Monitoring Board (DSMB), consisting of a clinical expert, a statistician, and an ethicist. The DSMB will be charged with providing guidance regarding subject recruitment and retention, issues related to implementation of the study protocol, any adverse events, and any complaints or problems emanating from participants. The DSMB will meet at least annually to review study progress and make recommendations to the investigators.

## Discussion

An increased QT_C _interval in heart transplant recipients is linked to acute allograft rejection and death. Normal variation in QT_C _intervals over 24 h due to changing autonomic nervous system tone does not occur in heart transplant recipients because transplant surgery causes denervation of the allograft. Thus, an increase in QT_C _is likely to indicate cellular dysfunction that occurs with acute rejection. Prior research is inadequate to determine whether monitoring the QT interval would be valuable to detect acute allograft rejection because the studies have been retrospective and involved just a few ECGs recorded periodically and analyzed by investigators who were not blinded from information about rejection episodes.

The NEW HEART study focuses on the potential for future reduction in the number of EMBs which transplant recipients must endure in the first year following transplantation. While EMB remains the gold standard for detection of cellular rejection in heart transplant recipients, it is costly, inconvenient, and not without risk [4,5,26]. Increased QT_C _has been linked to acute allograft rejection, but has not been systematically evaluated in prospective studies. This investigation will provide "real world" prospective data to determine the sensitivity and specificity of QT_C _as an early non invasive marker of cellular rejection in transplant recipients during the first post-transplant year.

A non-invasive indicator of early allograft rejection in heart transplant recipients has the potential to limit the number and severity of rejection episodes by reducing the time and cost of rejection surveillance and by shortening the time to recognition of rejection. Additionally, achievement of the aims of the current study may identify other ECG parameters relevant for non-invasive allograft rejection monitoring and may provide support for a randomized controlled trial to determine the efficacy and cost-effectiveness of this type of noninvasive ECG monitoring compared with standard EMB surveillance.

## Abbreviations

EMB: Endomyocardial Biopsy; ECG: Electrocardiogram; NEW HEART: Novel Evaluation with Home Electrocardiogram And Remote Transmission: CHARM Computerized Heart Allograft Recipient Monitoring; UCSF: University of California - San Francisco; SIM: Subscriber Identity Module; GPRS: General Packet Radio Services; CU-NYP: Columbia University-New York Presbyterian Medical Center; UCLA: University of California: Los Angeles (UCLA); CSMC: Cedars Sinai Medical Center; OAAIS: Office of Academic and Administrative Information Systems (OAAIS); VPN: Virtual Private Network; REDCap: Research Electronic Data Capture; DSMB: Data Safety Monitoring Board; QTc: QT interval corrected for heart rate.

## Competing interests

The authors declare that they have no competing interests.

## Authors' contributions

BD and LD developed the study concept, aims, and methods. BD, LD, and KH co-wrote the study protocol. All authors are implementing the study protocol. LD, KH, and BC are overseeing enrollment and collection of clinical data. BD and DP are overseeing electronic ECG data collection and analysis. BD, LD and KH will oversee final data analysis. LD and BC drafted the study manuscript and all authors contributed to, read and approved the final manuscript.

## Pre-publication history

The pre-publication history for this paper can be accessed here:

http://www.biomedcentral.com/1471-2261/12/14/prepub
